# Effects of Serum Incubation on Lipid Nanoparticle PEG Shedding, mRNA Retention, and Membrane Interactions

**DOI:** 10.1021/acsami.5c17052

**Published:** 2025-11-14

**Authors:** Simon Niederkofler, Petteri Parkkila, Nima Aliakbarinodehi, Nima Sasanian, Gustav Emilsson, David Ulkoski, Celso J.O. Ferreira, Nicole Stéphanie Galenkamp, Bruno F.B. Silva, Dan Lundberg, Yujia Jing, Lennart Lindfors, Björn Agnarsson, Fredrik Höök

**Affiliations:** 1 Department of Physics, Division of Nano and Biophysics, 11248 Chalmers University of Technology, Fysikgränd 3, Göteborg 41296, Sweden; 2 Advanced Drug Delivery, Pharmaceutical Sciences, BioPharmaceuticals Research and Development, 128698 AstraZeneca, Gothenburg 431 83, Sweden; 3 Advanced Drug Delivery, Pharmaceutical Sciences, BioPharmaceuticals Research and Development, AstraZeneca Boston, Waltham, Massachusetts 02451, United States; 4 INL-International Iberian Nanotechnology Laboratory, Braga 4715-330, Portugal; 5 Department of Chemistry, Physical Chemistry, Lund University, Naturvetarvägen 14, Lund 22100, Sweden; 6 Center for X-Ray Analytics, Laboratory for Biointerfaces, and Laboratory for Biomimetic Membranes and Textiles, Empa, Swiss Federal Laboratories for Materials Science and Technology, St. Gallen 9014, Switzerland

**Keywords:** lipid nanoparticle (LNP), mRNA delivery, endosomal escape, endosomal membrane mimic, protein corona, PEG shedding

## Abstract

Lipid nanoparticles (LNPs) are widely used for RNA delivery, but their efficiency remains limited, largely due to poor endosomal escape. Upon administration, proteins bind to the surface of the LNPs, influencing cellular uptake and potentially altering their interfacial properties. Such alterations may also affect their interaction with endosomal membranes, thus influencing the critical endosomal escape step. Using fluorescence microscopy imaging with single-LNP resolution, this study investigates how incubation in 10% fetal bovine serum alters the PEG modification and mRNA content of LNPs, as well as how serum incubation-induced alterations influence the interaction between LNPs and an anionic supported lipid bilayer (SLB), serving as a simplistic mimic for the anionic lipid membrane of late endosomes. We demonstrate that serum incubation leads to the desorption of PEG-modified lipids and a significant release of mRNA cargo from the LNPs. PEG shedding occurred consistently with a half-life time of around 10 min, while mRNA release displayed higher variability between individual LNPs. We also observed that serum preincubation enhanced attractive interactions between tethered LNPs and the anionic SLB at physiological pH 7.4, and fusion of LNPs with the anionic SLB upon pH reduction was more efficient for serum-preincubated LNPs than for their pristine counterparts, particularly during moderate acidification from pH 6.5 to 6.0. This enhanced fusion efficiency may be attributed to a reduced steric hindrance from PEG-lipids following serum preincubation. The findings highlight that serum-induced modifications enhance LNP fusion efficiency with an endosomal membrane mimic while potentially compromising mRNA retention, thus balancing the overall efficacy of LNP-assisted mRNA delivery.

## Graphical abstract

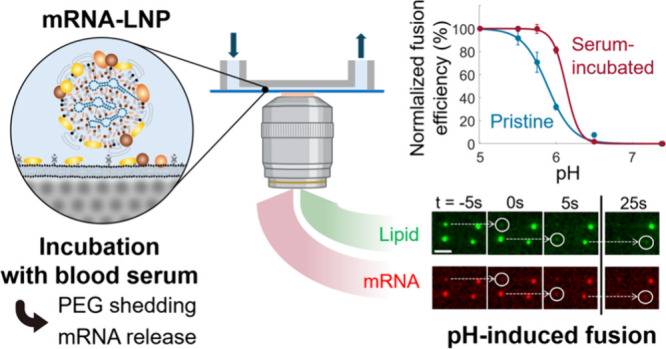

## Introduction

Translating mRNA-based therapies into clinical practice, particularly in protein replacement applications, requires the development of vehicles that ensure efficient and safe transport of these highly charged, high-molecular-weight therapeutics into target cells.^1–3^ Lipid nanoparticles (LNPs) have emerged as the most successful nonviral delivery platform for nucleic acids, underpinned by advances such as FDA-approved mRNA vaccines^4,5^ for COVID-19 and RNA interference therapies such as patisiran.^6^

These achievements underscore the potential of LNPs for endocytic uptake and successful mRNA-assisted protein expression, which is currently being adapted for targeted cell delivery reaching beyond vaccine applications.^7^ However, despite advancements in targeting specific cells,^8^ the transfection efficiency of LNPs remains significantly lower than that of viral vectors,^9^ with the main challenge being related to mRNA translocation across the endosomal membrane after LNPs are endocytosed.^10–12^

The so-called endosomal escape step that is required for successful mRNA release into the cellular cytosol is facilitated by the gradual acidification of the endosomal lumen, which is considered to lead to a lipid phase transition in the LNPs induced by protonation of their ionizable lipids.^13–15^ In addition, the protonated ionizable lipids are expected to promote electrostatic attraction between the LNP and anionic endosomal membranes,^16,17^ which, combined with the lipid phase transition, results in LNP disintegration and subsequent mRNA translocation across the endosomal membrane.^18,19^ However, functional delivery of nucleic acids is inefficient; for instance, less than 2% of endocytosed low-molecular-weight siRNA cargos induce a functional response^20,21^ with even lower efficiencies reported for mRNA.^22^

In addition to the cationic ionizable lipid, which is being utilized both to encapsulate nucleic acids and to facilitate their intracellular delivery, LNPs are composed of “helper lipids” such as phospholipids, sterols, and PEGylated lipids. PEG-lipids influence key quality attributes of LNPs, such as average size, size dispersity, and storage stability.^23^ Once injected, they also influence delivery efficiency by affecting circulation time and uptake by target cells.^24^ To balance sufficient circulation lifetime with cellular delivery, the PEG moiety is typically conjugated to phospholipids or glycerolipids with 14-carbon tails, which promote PEG-lipid desorption upon administration,^25^ which has been demonstrated to improve LNP-based delivery to the liver.^26^

PEG-lipid desorption, also called PEG shedding, is intrinsically linked to the interaction of LNPs with serum proteins. As LNPs enter circulation, serum proteins induce both desorption of PEG-lipids and the formation of a layer of adsorbed proteins on the LNP surface, a so-called protein corona.^27^ The formation and dynamic evolution of the protein corona composition, which is abundant in albumin, immunoglobulins, complement factors, and lipoproteins,^28,29^ give the LNPs a distinct biological identity,^30^ which in turn affects biodistribution, cellular uptake, and even preferential organ and cellular targeting.^8^

Since protein corona formation is inevitable upon LNP administration and also promotes cellular uptake, for example through ApoE-mediated endocytosis,^31^ corona proteins are expected to remain associated with LNPs throughout the endocytic process. As protein coronation will change the LNP surface identity and may also induce structural rearrangements,^32^ it is likely to affect the interaction of LNPs with the endosomal membrane. Given the critical role of LNP composition and structure for effective mRNA delivery,^33^ protein coronation could thus impact the efficiency of mRNA translocation into the cytosol, ultimately impacting the overall efficacy of the delivery system.

While the impact of protein coronation on LNP biodistribution and cellular uptake has been extensively studied,^34–38^ its effect on the endosomal escape process remains less explored. However, since protein corona formation on LNPs is required for spontaneous cellular uptake, the impact of protein corona formation on the interaction between LNPs and endosomal membrane is highly challenging to investigate in living cells.^39^ Yet, it has been shown that protein coronation can significantly influence interactions between nanoparticles and supported lipid bilayers (SLBs) serving as mimics of cellular membranes.^17,40–42^

Specifically, the presence of a protein corona has been shown to reduce pH-induced electrostatic binding of ionizable-lipid-containing LNPs to an anionic SLB,^17^ suggesting that protein coronation may negatively impact the capacity of LNPs to fuse and disintegrate with the endosomal membrane during gradual acidification within the endosome. Building on this observation, this study aims to extend recent advances^19,43^ that enable continuous, time-resolved fluorescence imaging of pH-induced fusion of LNPs with an anionic nanoporous-silica-supported lipid bilayer to assess the impact of protein coronation on this fusion process by including a serum preincubation step. Further, this study also explores the effects of serum preincubation on LNPs, quantifying PEG shedding kinetics and mRNA retention dynamics at the single-particle level. The results contribute to an improved understanding of LNP protein coronation and its potential impact on the endosomal escape process, which may provide relevant insights into the future design of LNPs.

## Results and Discussion

LNPs were preincubated in a medium containing 10% (v/v) fetal bovine serum (FBS), representative for a serum-containing medium used for in vitro cellular studies.^22,44^ To match the time required for efficient cellular uptake of serum-preincubated LNPs,^38^ the serum preincubation was performed for 3 h at room temperature, at an LNP concentration corresponding to ∼5 μg/mL mRNA, using citrate-phosphate buffer with pH 7.4 as a diluent. To assess LNP sample integrity, the hydrodynamic diameter of the serum-preincubated LNPs was compared with pristine LNPs, which were incubated under identical conditions but in the absence of FBS. The hydrodynamic diameter distribution was measured using nanoparticle tracking analysis (NTA), for which the serum-preincubated LNPs were diluted from 10 to 0.1% (v/v) FBS concentration within 5 min prior to the measurement. To account for the presence of biological nanoparticles in FBS, that overlap in size with the LNPs,^45^ the data for serum-preincubated LNPs were corrected by subtracting the size distribution measured in a 0.1% (v/v) FBS blank sample. Means and standard deviations of the obtained LNP diameter distributions, extracted by Gaussian fitting, showed minor differences of 138 ± 45 nm for pristine LNPs compared to 131 ± 41 nm for serum-treated LNPs (Figure 1a). Complementary cryo-electron microscopy analysis yielded consistent results, indicating that the applied serum preincubation conditions only have a minor effect on particle size but rather induce changes in particle structure (Figure S1).

**Figure 1:**
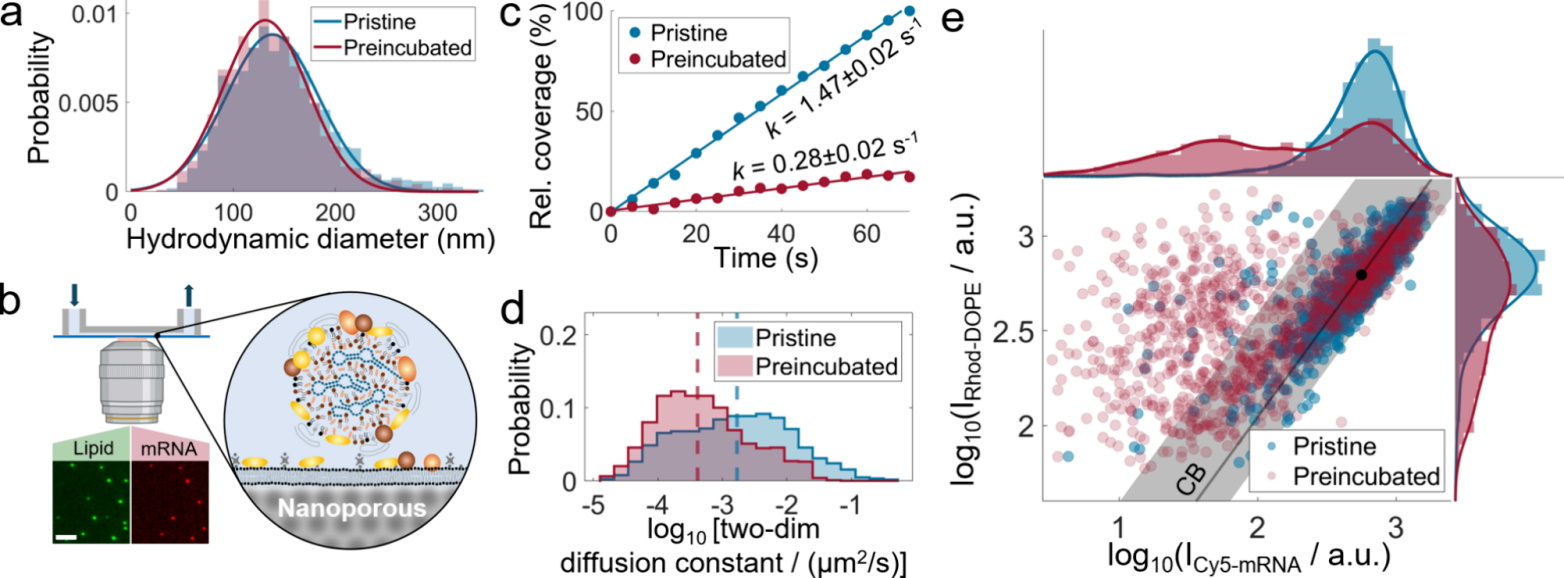
Effects of serum preincubation on lipid nanoparticle (LNP) size, their interaction with an anionic supported lipid bilayer (SLB), and mRNA content. (a) Hydrodynamic diameter distribution determined with nanoparticle tracking analysis of LNPs incubated for 3 h at room temperature in buffer (pristine) or 10% (v/v) fetal bovine serum (preincubated). Preincubated data were corrected for the presence of serum nanoparticles by subtraction of blank data. Means and standard deviations of 138 ± 45 and 131 ± 41 nm for pristine and preincubated LNPs, respectively, were determined using Gaussian fitting. (b) Schematic of the experimental setup used to study LNP-SLB interactions. Fluorescence microscopy imaging (scale bar = 5 μm) of LNPs immobilized on nanoporous-silica-SLB located on the floor of a microfluidic channel. (c) Relative coverage during NeutrAvidin–biotin-mediated tethering of pristine or serum-preincubated LNPs to SLB (both normalized with final pristine LNP coverage). Serum preincubation results in a lower binding rate *k*. (d) Distribution of the 2D diffusion constant *D* of tethered LNPs. Serum preincubation results in a shift to lower diffusivities, indicated by a median (dashed lines) of log_10_[*D*/(μm^2^/s)] = −2.77 for pristine LNPs (*n* = 229 LNPs) and −3.38 for preincubated LNPs (*n* = 401 LNPs). Data from single experiments with comparable coverages after tethering of ∼0.014 and ∼0.024 LNPs/μm^2^, respectively. (e) Log–log plot of the single-particle fluorescence intensity *I* (labeled lipid and mRNA moieties: Rhod-DOPE and Cy5-mRNA) of tethered LNPs showing a significant difference between pristine and preincubated LNPs (pooled from three experimental replicates, number of LNPs = 1204 for both data sets). Pristine LNPs scale close to unity (black line), while 32% of preincubated LNPs are outside of the 95% confidence band (CB) of pristine LNPs (shaded area; estimated based on spread around the unity line). The black dot represents the median Rhod-DOPE signal of pristine LNPs, projected on the unity line.

To investigate the impact of serum preincubation on the interaction of molecularly tethered LNPs with an anionic lipid membrane, a supported lipid bilayer was formed via adsorption and rupture of small unilamellar vesicles on a nanoporous silica film with a pore diameter of 6 nm, integrated into a microfluidic channel as described previously.^17^ The anionic SLB was prepared using lipid vesicles with a composition of POPC, BMP, DOPE-NBD, and DOPE-Cap-biotin at mole percentages of 89.7, 10, 0.25, and 0.05%, respectively (see Materials and Methods). The lipid BMP, prevalently found in late endosomal membranes,^46^ was chosen to mimic the negative charge of endosomal membranes. This selection replaces the previously utilized phosphatidylserine lipid,^19^ which undergoes protonation at pH levels below 6,^47^ making it less suitable for accurately representing the gradual pH reduction during the endosomal maturation process. The formation of a continuous SLB was verified using fluorescence recovery after photobleaching (FRAP),^48^ which revealed an immobile fraction of 0.09 ± 0.04 and a two-dimensional diffusion constant, *D*, of 3.2 ± 0.2 μm^2^/s (*n* > 3 replicates), being consistent with previously observed increases in lateral diffusivity of SLBs formed on nanoporous silica substrates compared to SLBs formed on planar glass.^49,50^ The DOPE-Cap-biotin containing SLB was subsequently incubated with NeutrAvidin (20 μg/mL, 10 μL/min for 10 min), followed by binding of either pristine or serum-preincubated LNPs at a concentration of ∼5 μg/mL mRNA (∼1 × 10^11^ LNPs/mL) at a volumetric flow rate of 5 μL/min.

The microfluidic-controlled immobilization of the fluorescently labeled LNPs was monitored by using time-resolved TIRF microscopy (Figure 1b) and interrupted when a viable LNP coverage was reached. Serum-preincubated LNPs displayed a binding rate that was around five times lower than that of pristine LNPs (Figure 1c and Movie S1). This is primarily attributed to the presence of biotin in FBS,^51^ which can block NeutrAvidin binding sites on the SLB, as confirmed by a 3-fold decrease in the rate of pristine LNP binding to the NeutrAvidin-functionalized anionic SLB after incubation with 10% FBS (10 μL/min, 5 min) compared to conditions without serum preincubation (Figure S2). LNPs that remained tethered after microfluidic-assisted rinsing were imaged in time-resolved TIRF mode with a single-LNP resolution. Comparison of the lateral diffusivity^52^ of pristine and serum-preincubated LNPs, quantified as the two-dimensional diffusion constant extracted from individual LNP trajectories,^52^ at comparable surface coverages of 0.014 and 0.024 LNPs/μm^2^, respectively, revealed that serum-preincubated LNPs exhibit almost a 10-fold reduction in diffusivity compared to pristine LNPs (Figure 1d and Movie S2). This suggests that protein coronation introduces a weak, yet attractive, interaction with the anionic endosomal membrane mimic, potentially facilitated by serum-induced PEG shedding.

Analyzing the DOPE-Lissamine-Rhodamine (Rhod-DOPE) and Cy5-labeled eGFP (Cy5-mRNA) fluorescence signals from individual LNPs through a log–log scatter plot reveals that the majority of pristine LNPs follow a scaling law with a slope close to unity (Figure 1e). Under the assumption that the distribution of the fluorescence signal correlates with LNP size,^53^ a slope of one suggests that Rhod-DOPE and Cy5-mRNA incorporate into LNPs with identical size dependencies, likely due to electrostatic attraction between cationic ionized lipids and the anionic groups of both mRNA and Rhodamine during the LNP formulation step. Defining a 95% confidence band based on the deviation of data points from the unity scaling trend shows that the 5% fraction of LNPs that fall outside this band generally exhibits a reduced Cy5-mRNA signal, suggesting a lower mRNA content (Figure 1e). In contrast, a significant 32% fraction of serum-preincubated LNPs fall outside this 95% confidence band for unity scaling behavior, with the fluorescence signal distribution of serum-preincubated LNPs displaying a significantly larger spread in both the Rhod-DOPE and the Cy5-mRNA signals (Figure 1e). Given that photophysical effects are expected to be minimal for this type of Cy5-mRNA-LNP,^19^ the likely cause of this observation is protein coronation-induced mRNA release, also accompanied by some lipid release. Indeed, lipid transport proteins such as apolipoproteins exhibit high affinity for this type of LNP,^29^ and their adsorption has been reported to induce both internal and potentially interfacial structural changes of the LNP.^32^ Our results suggest that such alterations appear to reduce the capacity of the LNPs to retain their encapsulated mRNA, which is a critical factor in effective mRNA delivery.

It is worth noting that alterations of the LNPs induced by serum protein binding should be considered in the context of the stabilizing PEG-lipids, which self-assemble on the LNP surface to aid colloidal stability by sterically screening intermolecular interactions. The LNPs used in this study were stabilized using a PEG(2000) moiety conjugated to the 14-carbon tail phospholipid DMPE. This stabilizer, in contrast to PEG-lipids with 18-carbon tails, has been reported to be released upon contact with serum proteins.^25^ To further explore how serum preincubation affects the LNP PEG coating and a possible correlation to mRNA release, LNPs were fabricated with cargo composition identical to those analyzed above but with 50% of the DMPE-PEG(2000) lipids being replaced with fluorescently labeled DMPE-PEG(2000)-ATTO488 (ATTO488-PEG-lipid), synthesized as described in Supporting Information, Sect.1. To prevent the possible transfer of PEG-lipids to the SLB, the LNPs were in these experiments tethered using NeutrAvidin to a protein-repelling^54^ and fusion-resistant PLL-*g*-PEG-functionalized glass surface containing 5 mol % biotinylated PLL-*g*-PEG (Figure 2a). A log–log scatter representation of ATTO488-PEG-lipid versus Cy5-mRNA signal shows the adherence to a scaling law with a slope of 2/3, in contrast to a slope of unity observed for Rhod-DOPE versus Cy5-mRNA signal (Figure 2b). A slope of two-thirds is consistent with LNPs having an mRNA-loaded core, scaling with the LNP radius as *r* ^3^, with a PEG-lipid-enriched surface, scaling as *r* ^2^. A slope of unity, observed for Rhod-DOPE signal plotted versus Cy5-mRNA signal, indicates that both components exhibit the same size dependency (Figure 1e).

**Figure 2:**
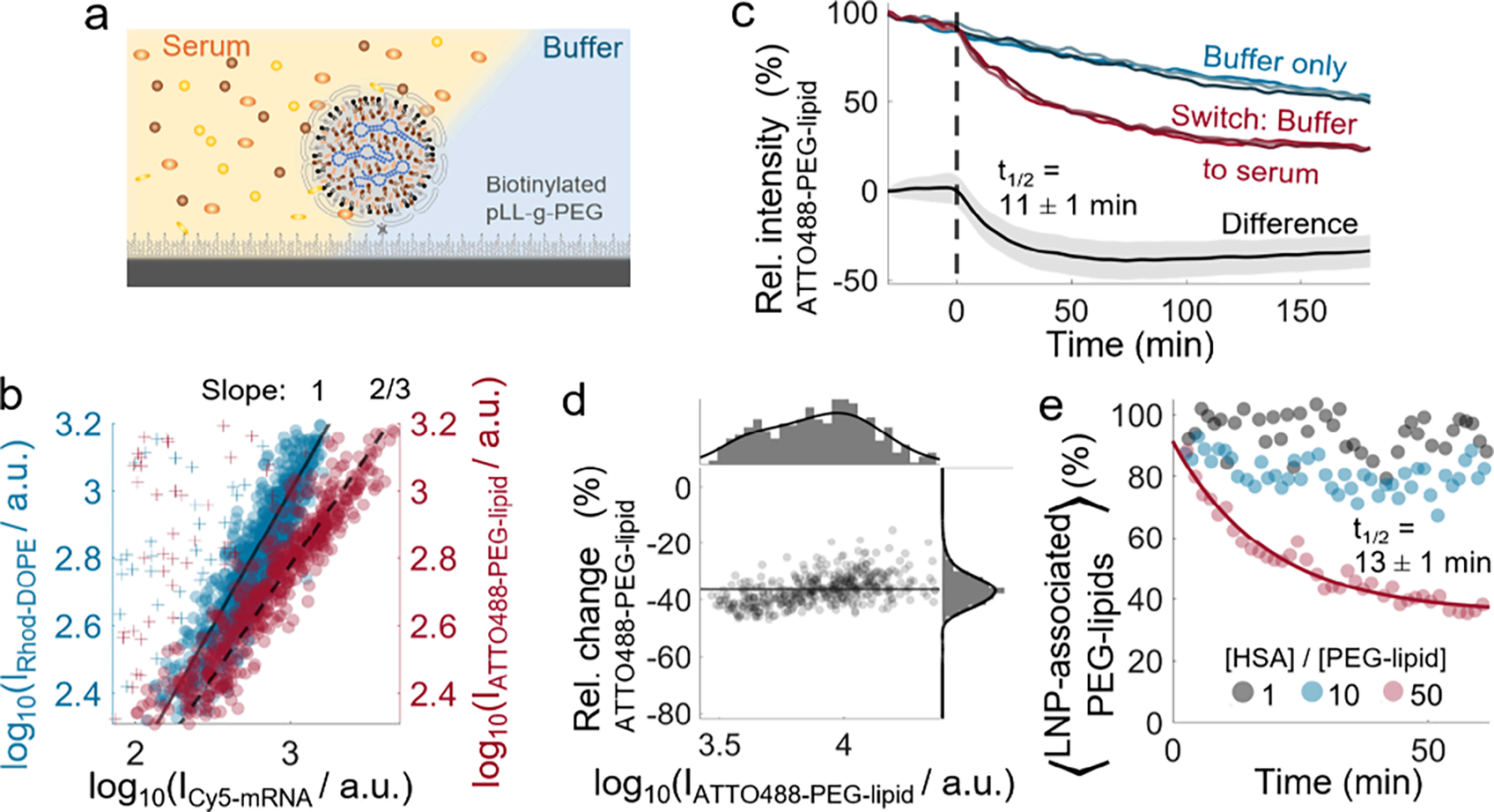
Time-resolved measurement of serum-induced PEG shedding from lipid nanoparticles (LNPs). (a) Schematic of the experimental setup. LNPs immobilized via NeutrAvidin–biotin tethering on a pLL-*g*-PEG-functionalized glass surface, enabling time-resolved imaging during microfluidic-assisted solution exchange. (b) Fluorescence signal *I* of individual LNPs labeled with ATTO488-PEG-lipid or lipid dye Rhod-DOPE as a function of their labeled mRNA (Cy5-mRNA) signal (crosses indicate LNPs with anomalously low Cy5-mRNA signal, representing 9 and 5% of the total number of observations, respectively). Scaling laws (slope = 1 or 2/3) indicate distinct mechanisms of dye incorporation for ATTO488-PEG-lipid and Rhod-DOPE. (c) Time-resolved fluorescence signal of the ATTO488-PEG-lipid for representative individual LNPs subjected to either buffer only (blue palette) or a buffer-to-serum switch at time *t* = 0 (red palette; dashed line indicating switch). The average signal difference between LNPs subjected to buffer only and to the buffer-to-serum switch (*n* = 158 and 454 LNPs, respectively) shows serum-induced PEG shedding (black line; shaded area ± 1 s.d.) with the half-life time (*t*_1/2_) and its uncertainty determined with an exponential fit on the average difference within 0 < *t* < 60 min. (d) PEG shedding after 180 min of serum incubation (quantified through the relative signal change) for individual LNPs (*n* = 454) as a function of their initial signal *I*. The average change of −36 ± 6% is indicated by a solid line. (e) Ensemble-averaged fraction of LNP-associated ATTO488-DMPE-PEG-lipids measured by fluorescence cross-correlation spectroscopy, using nonshedding Cy5-DSPE-PEG-lipids as an internal reference at varying molar ratios of human serum albumin (HSA) to total PEG-lipids. An exponential fit (red line) was applied to determine *t*_1/2_ at an HSA:PEG-lipid ratio of 50.

With a continuous flow of 20 μL/min of a pH 7.4 citrate-phosphate buffer, time-resolved measurements of the ATTO488-PEG-lipid signals of individual LNPs showed a continuous decline, while a notably enhanced signal decline was observed upon rapid switching (∼1 s for the field of view) to 10% FBS (Figure 2c and Movie S3). While the initial decline in pure buffer can be attributed to photobleaching, the accelerated decline observed when FBS is introduced suggests that the interactions with serum protein induce PEG shedding. By subtracting the ensemble-averaged signals of LNPs subjected to a buffer-only environment from those subjected to a switch to 10% FBS, we corrected the contribution of photobleaching. This correction reveals that pronounced PEG shedding occurs within the first tens of minutes of serum incubation and stagnates after approximately 60 min. A monoexponential fit with an offset yields a characteristic half-life of 11 ± 1 min for the PEG shedding (Figure 2c). Further, using the initial ATTO488-PEG-lipid signal as a proxy for the LNP size (Figure 2b), a scatter plot of the relative decrease in ATTO488-PEG-lipid signal versus the initial ATTO488-PEG-lipid signal for individual LNPs reveals an average PEG shedding of around 36 ± 6% and a slight, yet very small, higher PEG shedding in the lower size regime (Figure 2d). To verify that the observed PEG shedding was not influenced by immobilizing the LNPs on the sensor surface, fluorescence cross-correlation spectroscopy (FCCS) was used to investigate suspended LNPs of similar composition, incubated with different concentrations of human serum albumin (HSA). HSA was chosen as a representative serum protein due to its homology with BSA, the predominant protein in FBS, and its proposed role in PEG shedding.^44^ In addition, FBS shows considerable autofluorescence in the spectral range used for FCCS, which complicates the measurements and reduces the signal quality. The results revealed a similar PEG shedding rate of 13 ± 1 min at an HSA to PEG-lipid ratio of 50, corresponding to an HSA concentration (65 μM) similar to the albumin concentration of ∼30 μM in 10% FBS (information from supplier Gibco, Thermo Fisher Scientific) (Figure 2e). Interestingly, at lower HSA to PEG-lipid ratios of 1 and 10, PEG shedding is markedly reduced, indicating a strong concentration dependence of the shedding mechanism. Nonetheless, the FCCS measurements consistently support the identification of albumin as the primary serum component responsible for PEG shedding.^44^

Consistent with the reduction observed in the Cy5-mRNA signal for a subset of serum-preincubated LNPs (Figure 1e), analysis of the temporal change in Cy5-mRNA signal, measured concurrently with the ATTO488-PEG-lipid signal, revealed a pronounced 35 ± 27% mRNA release after 180 min of serum incubation compared to buffer-incubated counterparts upon switching from pH 7.4 buffer to 10% FBS (Figure 3a and Movie S4). While the ATTO488-PEG-lipid signal showed a monotonic decrease across all individual LNPs, the Cy5-mRNA signal decrease exhibited a higher variability in the rate of mRNA release, ranging from step-like to more gradual mRNA release (Figure 3a). Based on an automated detection of step-like mRNA release events, defined by a signal decrease rate greater than 5% per minute, 75% of LNPs showed a step-like release character. These releases occurred most frequently around 8 min after the start of serum exposure but were occasionally observed up to 180 min (Figure 3b).

**Figure 3:**
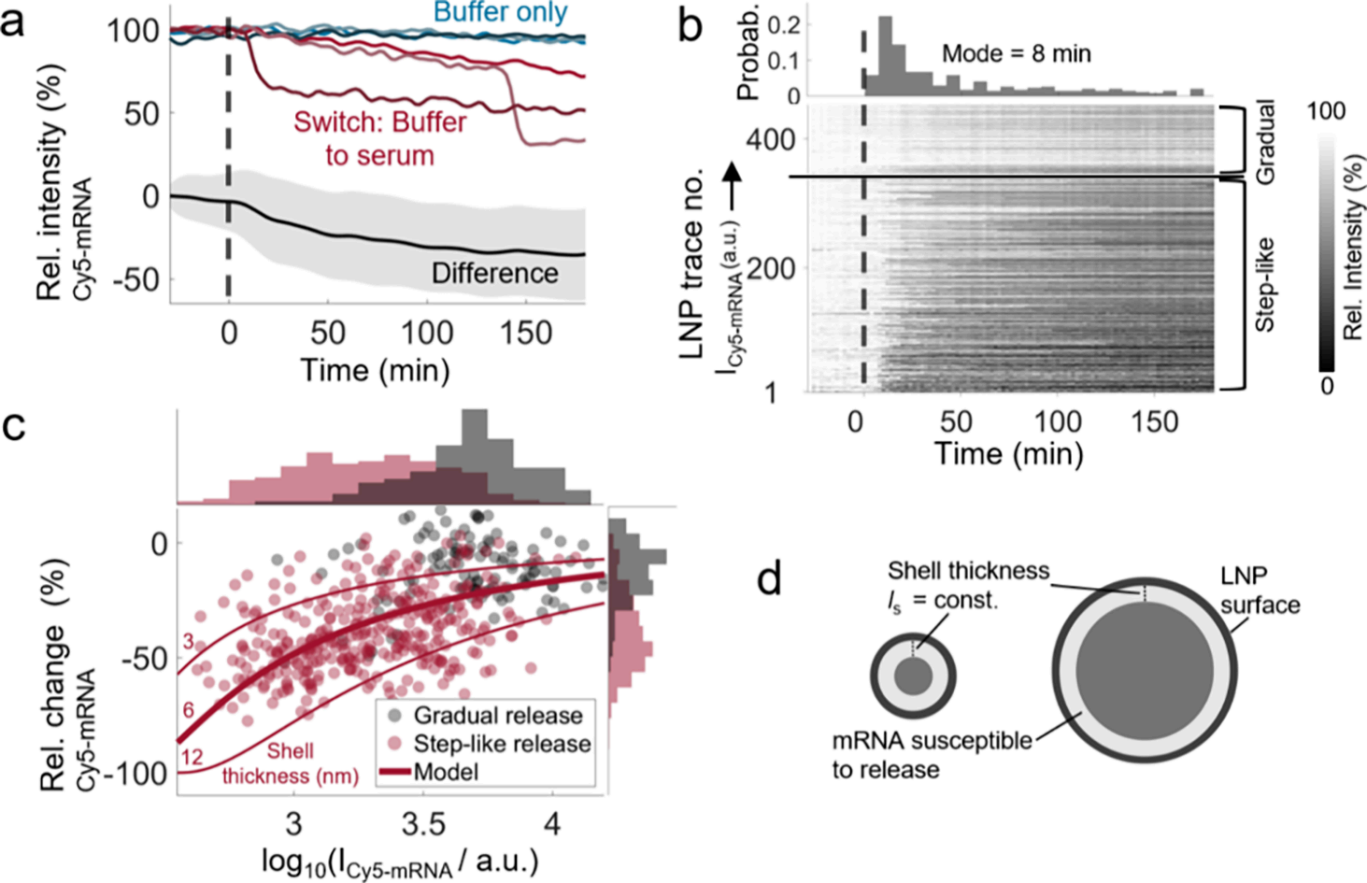
Time-resolved measurement of serum-induced mRNA release for lipid nanoparticles (LNPs). (a) Time-resolved fluorescence signal of Cy5-labeled eGFP-mRNA (Cy5-mRNA) for representative individual LNPs subjected to either buffer only (blue palette) or a buffer-to-serum switch at time *t* = 0 (red palette; dashed line indicating a switch). The average signal difference between LNPs subjected to buffer only and to the buffer-to-serum switch (*n* = 158 and 454 LNPs, respectively) shows serum-induced mRNA release (black line; shaded area ± 1 s.d.). (b) Kymograph visualizing the temporal Cy5-mRNA signal intensity variation of all individual LNPs upon exposure to serum at *t* = 0. Traces are categorized based on gradual or step-like release character (signal decrease rate greater than 5% per minute) and sorted in each category by their initial Cy5-mRNA signal. The histogram shows the distribution of step-like release times, peaking at 8 min after serum exposure. (c) mRNA release after 180 min of serum incubation (quantified through the relative signal change) for individual LNPs as a function of their initial signal *I* and distinction of LNPs displaying gradual or step-like mRNA release. A simplistic model compatible with the observed correlations (see main text) suggests that mRNA escapes from a shell with thickness *l*_s_ between 3 and 12 nm (red lines), as illustrated in (d).

Using the initial Cy5-mRNA signal as a proxy for LNP size (Figure 2b), a scatter plot of the relative Cy5-mRNA signal change demonstrates, in contrast to the PEG shedding, that smaller LNPs exhibit a higher relative release of mRNA compared with the larger counterparts (Figure 3c). Categorization of these data into gradual and step-like mRNA release events (Figure 3c, shown in black and red, respectively) revealed that step-like release is predominantly observed in smaller LNPs. This likely reflects the fact that the loss of a single mRNA molecule produces a larger relative signal decrease in smaller LNPs compared with larger ones.

A plausible explanation for the correlation between the extent of mRNA release and LNP size is that mRNA located within a certain distance *l*_s_ from the LNP surface is preferentially susceptible to protein coronation-induced release (Figure 3d). Under the assumption that *l*_s_ is independent of LNP radius *r*, the fraction Φ of mRNA located within this shell increases with a decreasing LNP radius as

$$\Phi \left(r\right)=1-{\left[\left(r-l_{s}\right)/r\right]}^{3}$$ 1

and consequently, the relative mRNA release increases with a decreasing LNP radius as −Φ­(*r*). By correlating the Cy5-mRNA signal prior to serum incubation with the LNP size distribution measured using NTA (Figure S3), a comparison of the data with this model (eq 1) shows consistency with mRNA escape from a region 3 to 12 nm beneath the LNP surface (Figure 3c, shown as red lines).

Assuming that the mRNA content scales proportionally with LNP volume, an LNP with a diameter of 140 nm encapsulates approximately 200 mRNA molecules.^33^ Correspondingly, an LNP in the smaller size regime (diameter of ∼80 nm, Figure 1a) would contain roughly 35 mRNA molecules, over half of which are released within the first 8 min of serum incubation. Such rapid mRNA release, occurring within a notably shorter duration compared to the timescale required for cellular LNP uptake,^22^ is anticipated to significantly impair the performance of LNPs, especially those with small-diameter and high-molecular-weight mRNA cargos.

Having validated that serum preincubation induces PEG shedding and partial mRNA release on a fusion-resistant interface, we now return to the LNPs tethered to the anionic SLB (Figure 1), which after tethering were exposed to sequential reductions in pH, aiming to represent the gradual acidification of the endosomal lumen. At pH 7.4, pristine LNPs exhibit a higher lateral diffusivity (∼1.8 × 10^–3^ μm^2^/s) compared to serum-preincubated LNPs (∼0.3 × 10^–3^ μm^2^/s), and there is only a moderate reduction in their lateral diffusivity when the pH is reduced from 7.4 via 6.5 (Figure 4a). In contrast, a significant reduction in diffusivity is observed for both pristine and serum-preincubated LNPs when the pH is further reduced to pH 6.0, with respective drops of 88 and 71% of the median value.

**Figure 4:**
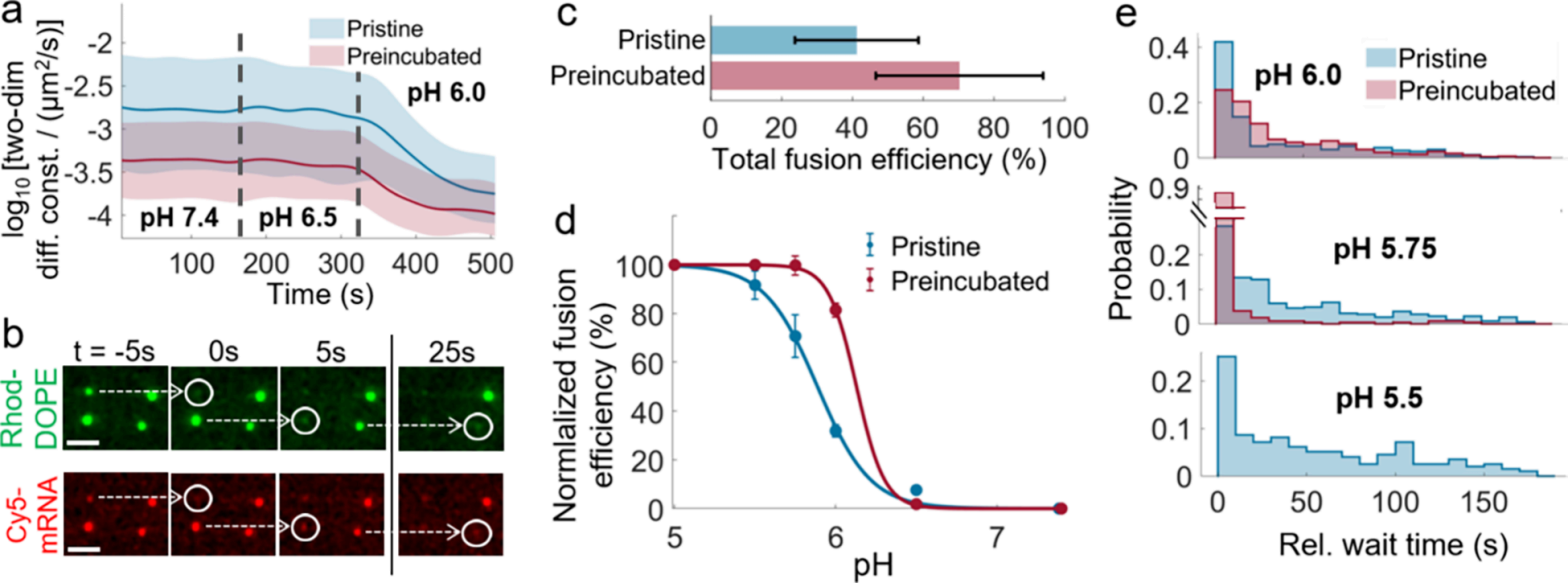
pH-induced fusion of pristine and serum-preincubated lipid nanoparticles (LNPs) with an anionic supported lipid bilayer (SLB). (a) pH decrease triggering the reduction of two-dimensional diffusion constants of LNPs tethered to anionic SLB. Distributions are shown with median (solid lines) and 25th to 75th percentile (shaded areas). At all measured pH values, serum-preincubated LNPs (>183 at any pH) exhibit lower average mobility compared to pristine LNPs (>248 at any pH). Data were extracted from nonfusing LNPs at each pH, based on single experiments with comparable coverages after tethering of ∼0.014 and ∼0.024 LNPs/μm^2^, respectively. (b) Fluorescence micrographs (Rhod-DOPE and Cy5-mRNA, representing labeled LNP lipids and cargos, respectively; scale bar = 2.5 μm) showing LNP fusion events triggered by a pH decrease from 6.5 to 6.0 (white circles). Fusion events display wait time relative to the first observed event (time *t* = 0), ranging from a few to several tens of seconds. (c) Total fusion efficiency extracted from three experimental replicates for pristine or serum-preincubated LNPs, assessed based on the cumulative percentage of fused LNPs at successive pH reductions (7.4, 6.5, 6.0, 5.75, 5.5, and 5.0), and (d) fusion efficiency as a function of pH, assessed based on the cumulative percentage of fused LNPs, normalized with total fusion efficiency. Solid lines represent sigmoidal fits to the data and uncertainty based on three replicates. (e) Relative wait time distributions for fusion events of pristine or serum-preincubated LNPs at a pH decrease from 6.5 to 6.0, 6.0 to 5.75, or 5.75 to 5.5 (data pooled from three replicates). Data for serum-preincubated LNPs at pH 5.5 are not shown due to the low event frequency.

The reduction in diffusivity between pH 6.5 and 6.0 coincides with the expected significant ionization of DLin-MC3-DMA-containing LNPs in this pH range,^19,55^ suggesting that electrostatic attraction to the anionic SLB caused by the presence of protonated ionizable lipids at the surface of the LNPs contributes to the drop in diffusivity. It cannot be excluded, though, that corona proteins also become protonated in this pH interval, potentially contributing to the 40% lower median diffusivity observed for serum-preincubated compared to pristine LNPs at pH 6.0.

The pronounced reduction in LNP mobility observed upon a reduction of the pH from 6.5 to 6.0 also coincides with LNPs starting to undergo fusion with the SLB, characterized by rapid (∼100 ms) disappearance of the Rhod-DOPE signal and a simultaneous reduction of the Cy5-mRNA signal (Figure 4b and Movies S5 and S6), in accordance with a previous study focused on pristine LNPs.^19^ This particular feature was investigated in a broader pH range to obtain the total fusion efficiency of LNPs by summing the fraction undergoing fusion at consecutive pH decreases from 7.4 to 6.5, 6.0, 5.75, 5.5, and 5.0, revealing that serum-preincubated LNPs exhibited an almost two times higher total fusion efficiency of 70 ± 23%, compared to 41 ± 17% for pristine LNPs (Figure 4c). It is also worth noting that a plot of fusion efficiency versus pH displays sigmoidal shapes with inflection points at pH 6.1 and 5.9 for serum-preincubated and pristine LNPs, respectively (Figure 4d), suggesting that serum preincubation facilitates LNP fusion at more moderate reductions of the pH.

The onset of LNP fusion was also quantified by measuring the wait times following the pH drop, with respect to the first observed fusion event, which typically occurred within seconds after rapid microfluidic liquid exchange (<1 s in the field of view). The wait times for pristine LNPs showed comparable distributions at pH 6.0, 5.75, and 5.5, with median wait times of 36, 44, and 55 s, respectively (Figure 4e). In contrast, serum-preincubated LNPs exhibited a median wait time of 39 s at pH 6.0, similar to pristine LNPs, while at pH 5.75, it was significantly shorter at 8 s (Figure 4e). Hence, the observed reduction in lateral diffusivity, increased fusion efficiency, and shorter wait time for serum-preincubated LNPs compared with pristine LNPs indicates that protein coronation promotes LNP fusion. This effect is likely related to reduced PEG-lipid-mediated steric hindrance following serum incubation. Furthermore, the electrostatically driven fusion could be facilitated by the presence of corona proteins with isoelectric points between 5.0 and 6.5,^56^ such as apolipoproteins.^57^

While serum preincubation appears to facilitate LNP fusion to the endosomal membrane at moderate pH, which is likely beneficial for functional mRNA escape, serum preincubation also resulted in unfavorable release of Cy5-mRNA, which motivates an analysis of whether LNPs with different Cy5-mRNA contents display different fusion efficiency. It is seen in Figure 5a that pristine LNPs fusing at pH 5.5 exhibit, on average, a 37% higher Cy5-mRNA signal and a 14% higher Rhod-DOPE signal compared to those fusing at pH 6.0. Assuming a positive correlation between fluorescence signal and size,^43^ this suggests that smaller LNPs require less acidification to undergo fusion. A similar but even more pronounced trend was observed for serum-preincubated LNPs. In particular, LNPs with significant reduction in fluorescence signals, characterized by reduced Cy5-mRNA and Rhod-DOPE signals after serum preincubation, exhibited preferential fusion at pH 6.0 (Figure 5b). Thus, although serum-induced alterations of the LNPs enhance the ability of LNPs to undergo pH-induced fusion at moderate acidification, a significant fraction (∼32%) of the fusion-competent LNPs at pH 6.0 also have a significantly lower Cy5-mRNA content compared to pristine LNPs. One plausible explanation for this observation is that protein coronation-induced mRNA escape leads to an increase in the fraction of ionizable lipids not engaged in complex formation with mRNA as the pH drops, which may, in turn, increase the electrostatic attraction to the anionic SLB.

**Figure 5:**
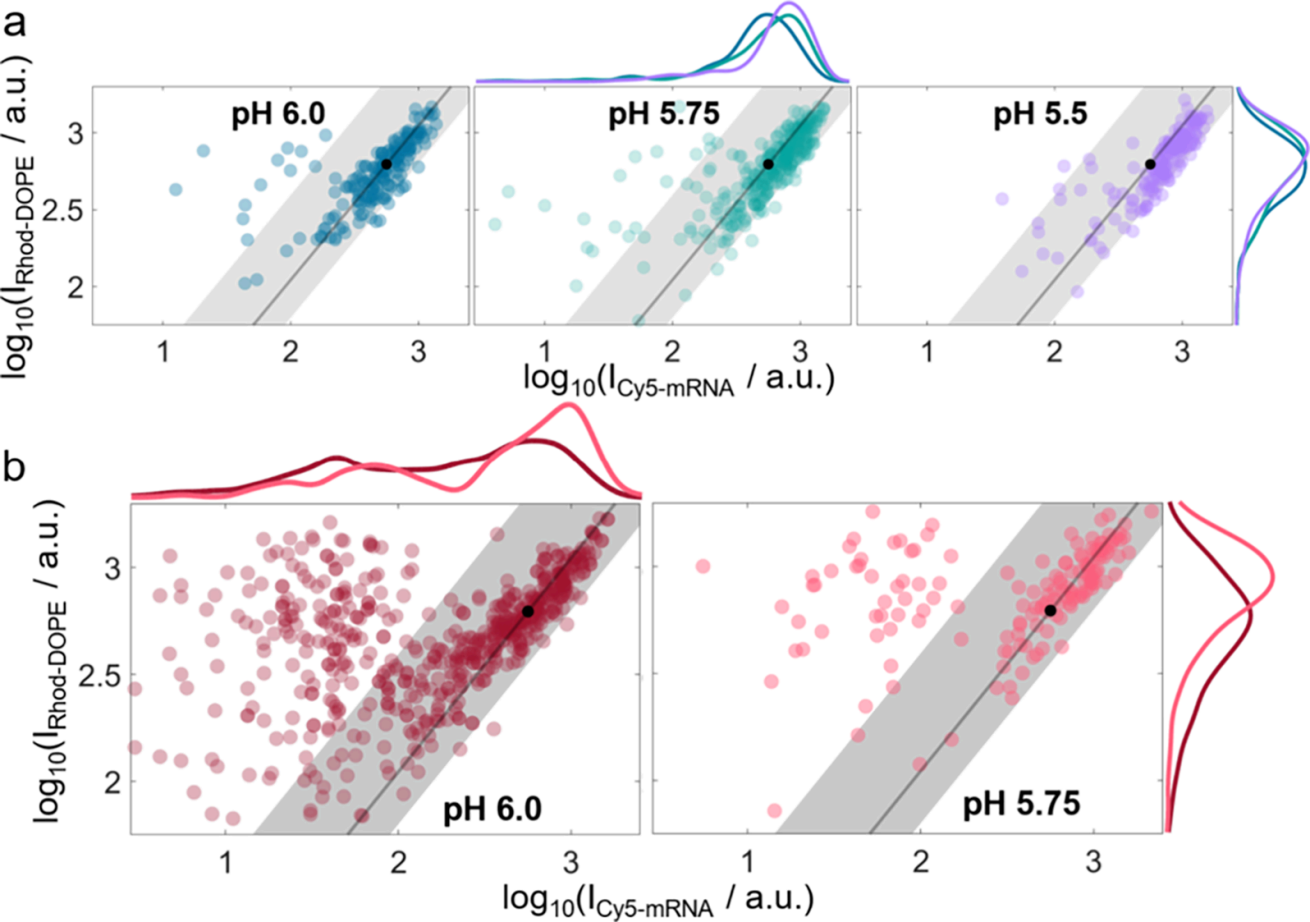
Log–log plot of the single-particle fluorescence signal of lipid nanoparticles (LNPs) undergoing pH-induced fusion with an anionic supported lipid bilayer (SLB). Fluorescence signals correspond to labeled lipid (Rhod-DOPE) and mRNA (Cy5-mRNA) moieties. (a) Pristine LNPs undergoing fusion upon pH decreases from 6.5 to 6.0 (*n* = 187 LNPs), 6.0 to 5.75 (*n* = 297 LNPs), or 5.75 to 5.5 (*n* = 168 LNPs). Data are pooled from three experimental repetitions. The solid line represents the unity scaling law, the black dot indicates the median Rhod-DOPE signal projected onto the unity line, and the shaded area shows the 95% confidence band (CB) for pristine LNPs at pH 7.4 (Figure 1e). (b) Serum-preincubated LNPs undergoing fusion upon pH decreases from 6.5 to 6.0 (*n* = 568 LNPs) and 6.0 to 5.75 (*n* = 151 LNPs). Data for the pH decrease from 5.75 to 5.5 are not shown due to the low number of observations. The solid line, black dot, and shaded area represent the unity scaling law, median Rhod-DOPE signal, and 95% CB for pristine LNPs at pH 7.4, respectively, as in (a). Data are pooled from three experimental repetitions.

## Conclusions

Our investigation demonstrates that serum incubation has a significant influence on the composition and properties of LNPs, affecting the PEG-lipid content and mRNA retention. While the hydrodynamic size of the LNPs remained essentially unchanged after 3 h of incubation in 10% FBS, notable changes were detected in the distribution of Rhod-DOPE and Cy5-mRNA signals across LNP size, substantiating effects of serum components on the LNP structure and mRNA payload. For pristine LNPs, the Rhod-DOPE and Cy5-mRNA fluorescence signals were observed to scale with particle volume, whereas notable deviations from this scaling trend arose after serum preincubation, indicating that fluorescence readout is not a reliable size estimator for LNPs after serum exposure. Additionally, the lack of a significant size change measured by NTA does not necessarily indicate the absence of substantial structural alterations in the LNPs.

Using single-particle fluorescence imaging, it was observed that approximately 36 ± 6% of the PEG-lipids detached from the LNPs, with a half life of roughly 10 min. Lower temperature^58^ and serum albumin concentration in 10% FBS compared to physiological conditions^59^ may contribute to the observed finite release of PEG-lipids. Serum incubation also induced substantial, albeit more variable, mRNA release of 35 ± 27%, with one fraction of LNPs exhibiting gradual mRNA release and another fraction step-like release. While PEG shedding displayed only a subtle dependence on LNP size, our results suggest that serum-induced mRNA loss is more pronounced in smaller LNPs, presumably because a larger proportion of the encapsulated mRNA is situated near the surface, which is most affected by serum protein binding. The findings are consistent with preferential mRNA release from an outer shell region of the LNPs, with an estimated shell thickness ranging from 3 to 12 nm, which corresponds to a size scale where interactions with serum proteins can plausibly be expected. With most release events occurring on a timescale faster than that expected for cellular LNP uptake, serum-induced mRNA release is unlikely to contribute to functional delivery, even if the released mRNA retains its structural integrity.

We also found that serum preincubation enhances interfacial interactions between LNPs and anionic SLBs at physiological pH, potentially facilitated by serum-induced PEG shedding. Additionally, upon stepwise reduction of the pH from 7.4 to 5.0, serum-preincubated LNPs were observed to display enhanced efficiency for pH-induced fusion with an anionic SLB, especially at more moderate pH 6.0 and 5.75 acidification. This observation is puzzling in the context of prior findings, suggesting that serum preincubation reduces electrostatically mediated binding of LNPs to an anionic SLB.^17^ However, we note that the molecular tethering used in this work extends the interaction time between LNPs and SLB, possibly enabling contributions of short-range and weak intermolecular interactions.^60^ Further, the rather significant PEG shedding observed upon serum preincubation (Figure 2) is expected to decrease steric screening of intermolecular forces between LNPs and anionic SLB and, as such, is likely a contributing factor for the observed lower diffusivity of serum-preincubated LNPs, as well as the increased propensity to undergo pH-induced fusion with the anionic SLB.

In conclusion, although serum preincubation has been reported to reduce the binding capacity of LNPs to an anionic SLB,^17^ the molecular tethering designed to mimic close contact between LNPs and the endosomal membrane after receptor-mediated endocytosis promotes pH-induced fusion of LNPs with an anionic SLB. The observed serum-induced effects on LNP composition and fusion propensity thus suggest that serum preconditioning may facilitate the release of therapeutic payloads under endosomal conditions, potentially enhancing the efficacy of LNP-based delivery systems. However, serum preincubation also leads to significant impairment of mRNA retention, which could potentially mitigate the positive effects. Thus, we speculate that the most successful LNP formulations *in vivo* will be those that strike a good balance between the retention of mRNA during circulation and enhanced release from the endosomes.

## Materials and Methods

### Lipid Nanoparticle Synthesis and Characterization

The LNPs were formulated using the microfluidic mixing method.^61^ In brief, the lipids DLin-MC3-DMA, cholesterol, DSPC, DMPE-PEG(2000), DOPE-Lissamine-Rhodamine, and DSPE-PEG(2000)-biotin were dissolved at mole percentages of 53.47, 41.114, 4.65, 0.7, 0.06, and 0.006 in ethanol, respectively, with a total lipid concentration of 7.0 mg/mL. For LNPs containing ATTO488-PEG-lipids, 0.35 mol % of DMPE-PEG(2000) was replaced with DMPE-PEG(2000)-ATTO488 (synthesized at AstraZeneca; more information in Supporting Information, Sect.1). The mRNA solution was prepared by diluting eGFP-mRNA and Cy5-labeled eGFP-mRNA at mole percentages of 80 and 20 (both purchased from TriLink), respectively, using RNase-free 100 mM citrate buffer pH 3.0 (Teknova) to obtain a total mRNA concentration of 0.23 mg/mL and a final citrate concentration of 50 mM. The mRNA and lipid solution were mixed in a 3:1 volume ratio at a flow rate of 12 mL/min using a NanoAssemblr benchtop device (Precision NanoSystems, Inc., Canada) microfluidic mixing device, yielding an LNP suspension with 1.75 mg/mL total lipid concentration and an mRNA:lipid weight ratio of 1:10 (DLin-MC3-DMA:nucleotide molar ratio ∼ 3:1). The LNP suspension was dialyzed overnight using a Slide-A-Lyzer G2 10K molecular weight cutoff dialysis cassette (Thermo Scientific) against a 500× sample volume of 8% (w/v) Tris-sucrose buffer. The LNP suspension was kept frozen at −80 °C until use. The encapsulation efficiency was determined using the RiboGreen assay (Thermo Fisher) for both Rhod-DOPE and ATTO488-PEG-lipid-labeled LNPs, resulting in efficiencies >90%. Due to the overlap in fluorescence emission of the RiboGreen dye and ATTO488, the encapsulation was estimated by performing a correction based on the fluorescence emission of a reference ATTO488-PEG-lipid LNP sample, which was not incubated with the RiboGreen dye. The LNP size was determined using nanoparticle tracking analysis (NTA) using a NanoSight LM10 device (Malvern Instruments) with a Hamamatsu C11440-50B/A11893-02 camera (Figure 1a and Figure S3).

For FCCS experiments specifically, LNPs were prepared using the same general procedure as described above with the lipid solution containing DLin-MC3-DMA, cholesterol, DSPC, DMPE-PEG(2000), DMPE-PEG(2000)-ATTO488, and DSPE-PEG(2000)-Cy5 at molar percentages of 50.0, 38.5, 10.0, 1.31, 0.11, and 0.08, respectively. The mRNA (0.25 mg/mL) and lipid (7.3 mg/mL) solutions were mixed in a 3:1 volume ratio, yielding an LNP suspension with a total lipid concentration of 1.83 mg/mL and a DLin-MC3-DMA:nucleotide molar ratio of ∼3:1. Dialysis was performed using a Slide-A-Lyzer G2 10K against a 300× sample volume of PBS (phosphate-buffered saline). LNPs were characterized regarding mRNA encapsulation (92%) and final mRNA concentration (0.075 mg/mL) using RiboGreen, as well as size (83 nm) using dynamic light scattering (ZetaSizer Nano, Malvern Instruments). Notably, the LNPs used in the FCCS experiments contained a higher total amount of PEG-lipids, resulting in a smaller average size compared to those in the main experiments, however maintaining the same density of PEG-lipids on the particle surface.^33^

### Nanoporous Silica Thin-Film Formation

Nanoporous silica thin films were synthesized by following a modified method by Alberius et al.^62^ Briefly, 0.28 g of poly­(ethylene glycol)-*block*-poly­(propylene glycol)-*block*-poly­(ethylene glycol) (P123, Sigma-Aldrich) was dissolved in 1.33 g of ethanol (99.5%, Solveco) in a glass vial. This mixture was stirred using a magnetic stirrer at room temperature until it was completely dissolved. In a separate vial, 1.73 g of tetraethylorthosilicate (TEOS, 98%, Sigma-Aldrich) and 2 g of ethanol were combined and stirred at 300 rpm with a magnetic stirrer. Subsequently, 0.9 g of 0.01 M HCl (Sigma-Aldrich) was added dropwise to this mixture, and the mixture was stirred continuously for 20 min. After this period, the P123 solution was mixed with the TEOS solution. This silica precursor solution was then stirred at room temperature at 300 rpm for 20 min to achieve a homogeneous and clear solution. The silica precursor solution was deposited onto borosilicate cover glasses (Menzel-Gläser, D263, no. 1) through spin coating at 4000 rpm (WS-650, Laurell Technologies Corporation). This was done immediately after submerging the glasses in an EtOH–NaOH (5:1) cleaning solution for 5 min, followed by a thorough rinse with ultrapure water and drying using nitrogen gas. The coated glasses were then left in the dark to age at room temperature for 24 h. The templating agent was removed by gradual heating at a rate of 1 °C/min from room temperature to 400 °C and maintaining this temperature for 4 h before allowing cooling to room temperature.

### Preparation of Supported Lipid Bilayers (SLBs)

Lipid vesicles were prepared by mixing the lipids 1-palmitoyl-2-oleoyl-*sn*-glycero-3-phosphocholine (POPC), *sn*-(3-(9*Z*-octadecenoyl)-2-hydroxy)-glycerol-1-phospho-*sn*-3′-(1′-(9*Z*-octadecenoyl)-2′-hydroxy)-glycerol (S,R Isomer) (BMP), 1,2-dioleoyl-*sn*-glycero-3-phosphoethanolamine-*N*-(biotinyl) (DOPE-Cap-biotin), and 1,2-dioleoyl-*sn*-glycero-3-phosphoethanolamine-*N*-(7-nitro-2–1,3-benzoxadiazol-4-yl) (DOPE-NBD) (all purchased from Avanti Research, USA), each dissolved in chloroform, to achieve the target composition. The solvent was removed by evaporation under a stream of nitrogen gas, followed by an additional 3 h drying step under vacuum. The resulting lipid film was rehydrated in PBS to a final lipid concentration of 2 mg/mL. The lipid suspension was then subjected to extrusion through a mini extruder (Avanti Polar Lipids, Inc., Alabaster, AL, USA) equipped with a 50 nm pore-size polycarbonate membrane (Whatman, Maidstone, UK). The vesicle suspension was passed through the membrane 25 times to obtain unilamellar vesicles of a uniform size. The prepared vesicles were stored at 4 °C until further use.

For experiments involving SLB formation, coverslips coated with nanoporous silica thin films were first cleaned by sequential rinsing with ultrapure water and 95% ethanol, followed by drying under nitrogen gas. The substrates were then treated with UV–O_3_ for 20 min. Immediately following the cleaning procedure, the substrates were assembled into sticky-Slide VI 0.15 microfluidic chambers (ibidi GmbH, Gräfelfing, Germany) and hydrated with PBS to form channels with length × width × height = 17 × 0.1 × 0.15 mm. SLBs were formed by introducing the lipid vesicle suspension (0.2 mg/mL, 50 μL/min, approximately 0.5 mL) into the microfluidic channel until a continuous SLB was established. The continuity and lateral fluidity of the SLB were confirmed by fluorescence recovery after photobleaching (FRAP). A defined circular region of the SLB was photobleached using a 532 nm laser (BWN Series of B&W Tek, Inc., Newark, USA). Fluorescence recovery was imaged at a frame rate of 0.2 fps. FRAP data were analyzed using a custom MATLAB script (MathWorks, Inc., USA).

### pLL-*g*-PEG Surface Functionalization

Glass slides (Menzel-Gläser, D263, no. 1) were thoroughly cleaned by sequential rinsing with ultrapure water and 95% ethanol, followed by drying with nitrogen gas. Subsequently, the slides were treated with UV–O_3_. Immediately following the cleaning procedure, the substrates were assembled into sticky-Slide VI 0.4 microfluidic chambers (ibidi GmbH, Gräfelfing, Germany) and hydrated with PBS to form channels with length × width × height = 17 × 4 × 0.4 mm. Surface functionalization was performed using a 5% biotinylated pLL-*g*-PEG mixture consisting of PLL(20)-*g*[3.5]-PEG­(2) and PLL(20)-*g*[3.5]-PEG­(2)/PEG­(3.4)-biotin­(50%) (SuSoS AG, Switzerland). The microfluidic channels were incubated under static (no-flow) conditions with a 100 μg/mL solution of the pLL-*g*-PEG for approximately 10 min. During the incubation, the solution was manually mixed twice. After incubation with pLL-*g*-PEG, the channel was rinsed with 5 mL of PBS. Subsequently, it was incubated with a 20 μg/mL NeutrAvidin solution for approximately 10 min, with manual mixing performed twice during the incubation. Finally, the channel was rinsed again with 5 mL of PBS to remove unbound NeutrAvidin.

### Fluorescence Microscopy

Microscopy imaging was performed at room temperature. The microfluidic system was mounted on an inverted Eclipse Ti-E microscope (Nikon Corporation, Minato City, Japan) equipped with a CFI Apo TIRF 60× (NA 1.49) oil immersion objective (Nikon Corporation, Tokyo, Japan). LNP fusion imaging was performed using TIRF illumination, utilizing a TRITC filter set for the Rhod-DOPE and a Cy5 ET filter set for Cy5-mRNA, while time-resolved imaging of serum incubation was performed using EPI illumination.

### Image Analysis

The positions of individual particles in the fluorescence micrographs were determined using threshold-based maxima detection, followed by a subpixel position determination employing radial symmetry characteristics.^63^ For time-resolved data, the particle positions were linked into trajectories using the Hungarian algorithm.^64^ The emission intensities were extracted from the background-subtracted micrographs as the sum of pixel values in a quadratic area, with the center defined by the position and the side length selected to reflect the average extension of particle signals in the micrographs.

A custom MATLAB code was used for the automated detection of LNP fusion events based on the signal change of Rhod-DOPE, which was verified and supplemented by visual event identification using the ImageJ PointPicker plugin (Philippe Thévenaz, Biomedical Imaging Group, Swiss Federal Institute of Technology, Lausanne). The identical principle was applied for the analysis of Cy5-mRNA signal changes upon serum incubation, where a threshold for the rate of change was used to detect step-like release events (cf. Figure 3).

### Particle Mobility Analysis

LNP mobility was quantified based on single-particle trajectories (*x*(*t*_*i*_), *y*(*t*_*i*_))_*i* = 1, ..., *n*_, which were obtained using a custom single-particle tracking algorithm. The two-dimensional diffusion coefficient, *D*, of each particle was calculated as the arithmetic mean of the one-dimensional diffusion coefficients, *D*_*x*_ and *D*_*y*_, along orthogonal spatial dimensions *x* and *y* in the micrographs. The one-dimensional diffusion coefficients, demonstrated here for the *x*-component, were obtained by measuring particle displacements Δ*x*(*t*_*i*_) over a time interval Δ*t* = *t*_*i*+1_ – *t*_*i*_, where Δ*x*(*t*_*i*_) = *x*(*t*_*i*+1_) – *x*(*t*_*i*_). The mean squared displacement (MSD) was calculated based on the variance of displacements, MSD*_*x*_* = ⟨[Δ*x*(*t*_*i*_) – ⟨Δ*x*(*t*_*i*_)⟩]^2^⟩ with *i* = 2, ..., *n*, from which the one-dimensional diffusion coefficient *D*_*x*_ = MSD/(2Δ*t*) was obtained.

### Fluorescence Cross-Correlation Spectroscopy (FCCS)

FCCS was used as a complementary technique to quantify the PEG shedding from LNPs following exposure to serum albumin. FCCS enables the detection of codiffusion of molecules or particles labeled with spectrally distinct fluorescent dyes, allowing quantification of the colocalization between DMPE-PEG(2000)-ATTO488 and DSPE-PEG(2000)-Cy5.^65,66^ Due to the much slower shedding kinetics of DSPE-PEG-lipids compared to DMPE-PEG-lipids,^44^ the Cy5 signal served as a reference marker for the LNPs.

More specifically, fluorescence intensity fluctuations yield autocorrelation and cross–correlation functions:^65^

G(τ)=A×D(τ) 2

where *D*(τ) is the diffusion component and *A* is the amplitude. For autocorrelation, *A* is inversely proportional to the number of fluorescent species in the confocal volume, and for cross-correlation, *A* is proportional to the number of colocalized fluorescent species. The measured amplitudes for the ATTO488, Cy5, and cross-correlation channels (denoted as *A*_A_, *A*_C_, and *A*_X_, respectively) can be modeled as

$$A_{A}=\frac{C_{P}+n^{2}\times C_{\mathrm{LNP}}}{V_{A}\times {\left(C_{P}+n\times C_{\mathrm{LNP}}\right)}^{2}}$$ 3a

$$A_{C}=\frac{1}{V_{C}\times C_{\mathrm{LNP}}}$$ 3b

$$A_{X}=\frac{n}{V_{X}\times \left(C_{P}+n\times C_{\mathrm{LNP}}\right)}$$ 3c

where *C*_P_ is the concentration of free PEG-lipid in solution, *C*_LNP_ is the concentration of LNPs, *n* is the average number of PEG-lipid molecules anchored per LNP, *V*_A_ and *V*_C_ are the effective volumes for the ATTO488 and Cy5 signal, respectively, and *V*_X_ is the overlap volume between *V*_A_ and *V*_C_. The ratio of cross-correlation to Cy5-channel amplitudes is

$$A_{X/C}=\frac{V_{C}\times n\times C_{\mathrm{LNP}}}{V_{X}\times \left(C_{P}+n\times C_{\mathrm{LNP}}\right)}$$ 4

This ratio reflects the fraction of ATTO488-PEG-lipids colocalized with LNPs. At the initial time point (*t* = 0), when all PEG-lipids are expected to remain associated with the LNPs, the ratio (*A*_X/C_)_*t* = 0_ serves as a reference for complete colocalization. The normalized time-dependent cross-correlation ratio is then

$$f_{\mathrm{LNP}‐\mathrm{associated}\;\mathrm{PEG}}\left(t\right)=\frac{{\left(A_{X/C}\right)}_{t}}{{\left(A_{X/C}\right)}_{t=0}}$$ 5

This normalization eliminates the dependence on the confocal volume ratio *V*_C_/*V*_X_.

Experiments were conducted using two silicon wells (ibidi, cat. no. 80209) mounted on a 170 ± 5 μm coverslip. LNPs were diluted to 5 μg/mL mRNA in either PBS or HSA (Alburex, CSL Behring) diluted in PBS. One well contained LNPs in PBS (control), and the other in HSA, with a coverslip placed on top to prevent evaporation. Control measurements were recorded before and after each kinetic series to exclude colocalization loss unrelated to HSA. Data acquisition began ∼90 s after HSA addition, using a Zeiss LSM 780-NLO confocal microscope, a C-Apochromat 40×/1.2 W objective, and 488 and 633 nm lasers. Each kinetic trace consisted of consecutive 5 s acquisitions, grouped into 30 s bins for correlation analysis. Individual 5 s segments deviating by >2 standard deviations in amplitude or count rate were excluded to remove aggregates or artifacts. Final kinetic traces were smoothed by averaging every three consecutive 30 s of *A*_X_/*A*_C_ values.

## Supplementary Material















## Notes

Synthesis of DMPE-PEG(2000)-ATTO488, impact of serum incubation on the size and morphology of lipid nanoparticles assessed by cryogenic transmission electron microscopy, effect of serum on NeutrAvidin–biotin-based lipid nanoparticle tethering, and size distribution of DMPE-PEG(2000)-ATTO488-labeled lipid nanoparticles (PDF)
NeutrAvidin–biotin-mediated binding of LNPs to SLB (Movie S1) (AVI)
Mobility of tethered LNPs at pH 7.4 (Movie S2) (AVI)
PEG shedding (Movie S3) (AVI)
mRNA release from immobilized LNPs upon serum incubation (including liposome reference particles, which were excluded from analysis) (Movie S4) (AVI)
pH-induced fusion of pristine LNPs (Movie S5) (AVI)
Serum-preincubated LNPs (Movie S6) (AVI)
