# Supplementary material for: Effects of Serum Incubation on Lipid Nanoparticle PEG Shedding, mRNA Retention, and Membrane Interactions
Source: ACS Appl Mater Interfaces. 2025 Nov 14;17(47):64219–31. doi: 10.1021/acsami.5c17052 (PMC12673520; doi:10.1021/acsami.5c17052)
Supplement: Supplementary file 1 [file am5c17052_si_001.pdf]

Supporting Information for:

Effects of Serum Incubation on Lipid Nanoparticle PEG Shedding, mRNA Retention, and Membrane Interactions

Simon Niederkofler[1,*], Petteri Parkkila[1], Nima Aliakbarinodehi[1], Nima Sasanian[1], Gustav Emilsson[2], David Ulkoski[3], Celso J.O. Ferreira[4], Nicole Stéphanie Galenkamp[5], Bruno F.B. Silva[4, 6], Dan Lundberg[2], Yujia Jing[2], Lennart Lindfors[2], Björn Agnarsson[1], and Fredrik Höök[1,*]

1) Chalmers University of Technology, Department of Physics, Division of Nano and Biophysics, Fysikgränd 3, 41296 Göteborg, Sweden
2) Advanced Drug Delivery, Pharmaceutical Sciences, BioPharmaceuticals Research and Development, AstraZeneca, Gothenburg ,Sweden
3) Advanced Drug Delivery, Pharmaceutical Sciences, BioPharmaceuticals Research and Development, AstraZeneca Boston, Waltham, MA 02451
4) INL-International Iberian Nanotechnology Laboratory, 4775-330 Braga, Portugal
5) Lund University, Department of Chemistry, Physical Chemistry, Naturvetarvägen 14, 22100 Lund, Sweden
6) Empa, Swiss Federal Laboratories for Materials Science and Technology, Center for X-Ray Analytics, Laboratory for Biointerfaces, and Laboratory for Biomimetic Membranes and Textiles, 9014 St. Gallen, Switzerland

* Corresponding authors

Email: fredrik.hook@chalmers.se, simon.niederkofler@chalmers.se

This PDF file includes:

1. Synthesis of DMPE-PEG2000-ATTO488 2
2. Impact of serum incubation on the size and morphology of lipid nanoparticles assessed by cryogenic transmission electron microscopy 3
3. Effect of Serum on NeutrAvidin-Biotin-based Lipid Nanoparticle Tethering 4
4. Size Distribution of DMPE-PEG(2000)-ATTO488-labeled Lipid Nanoparticles 4

1. Synthesis of DMPE-PEG2000-ATTO488

Materials and Reagents for the synthesis

1,2-Dimyristoyl-sn-glycero-3-phosphoethanolamine (DMPE) and tBoc-NH-PEG2000-CO2-NHS was obtained from NOF America Corporation (White Plains, New York). Atto488-NHS was obtained from ATTO-TEC GmbH (Siegen, Germany). Hydrogen chloride, dioxane, dimethyl sulfoxide (DMSO), N,N-Diisopropylethylamine (DIPEA), and dichloromethane (DCM) were obtained from Fisher Scientific (Vancouver, BC). All reagents were used without further purification

Synthesis procedure

Step A: Preparation of Boc-HN-PEG2000-DMPE

Boc-NH-PEG2000-CO2-NHS (0.867 g, 0.377 mmol, 1.2 mol) was added to a solution of solution of DMPE (0.2 g, 0.314 mmol, 1 mol) and DIPEA (0.166 g, 0.224 mL, 1.29 mmol, 4.1 mol) in DCM (10 mL). The resulting suspension was vigorously mixed at 25°C for 24 hours. Once the reaction mixture became completely clear and the solvent was rotary evaporated, and the residue taken up in acetonitrile (10 ml). After overnight storage at 4°C the solution was centrifuged to separate traces of insoluble unreacted DMPE and evaporated. The resulting residue was purified by flash chromatography, elution gradient 0 to 20% DCM in methanol to separation lipidated product from unreacted starting materials. Crude product fractions were concentrated under reduced pressure to dryness and taken through to the next synthetic step.

Step B: Preparation of amino-PEG2000-DMPE

Cleavage of the amino protecting group from Boc-HN-PEG2000-DMPE was carried out in 4 M HCl in dioxane for 4 hours. After removal of the solvent and all the volatiles were dried under vacuum, the product was quantitatively recovered in the form of a white solid. Disappearance of the tBu peak at 1.43-1.45 ppm and appearance of two separate peaks at 3.2-3.4 (br m, CH-NH, 2H) and 3.46 (m, CH-NH, 2H) instead of 3.25-3.35 (br m, CH-NH, 4H) indicated that the deprotection went to completion.

Step C: Synthesis of Atto488-PEG2000-DMPE

Amino-PEG2000-DMPE (0.1 g, 0.036 mmol, 1 mol) was added to a solution of solution of Atto488-NHS ester (0.050 g, 0.0432 mmol, 1.2 mol) and DIPEA (0.023 g, 0.050 mL, 0.177 mmol, 4.1 mol) in DMF (2 mL). The resulting suspension was vigorously mixed at 25°C for 24 hours. Once the reaction mixture was complete and the mixture was dried under high vacuum. The conjugate was purified by preparative RP-HPLC or SFC. Purity and identity of the peptide were checked by analytical RP-HPLC and electrospray mass spectrometry.

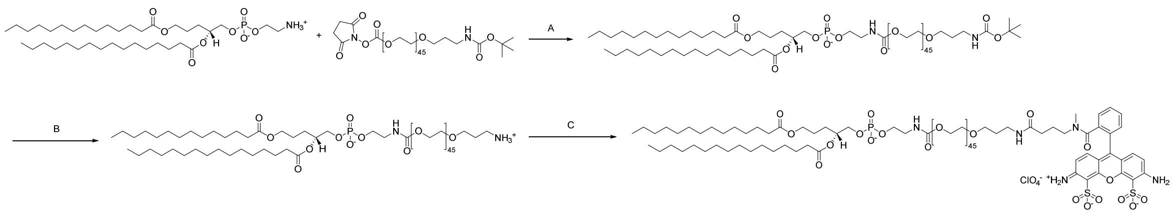

Scheme S1. Synthesis of DMPE-PEG2000-Atto488 accomplished through multistep synthesis: (A) DIPEA/DCM, (B) 1M HCl/Dioxane, (C) Atto488-NHS ester/DIPEA.

2. Impact of serum incubation on the size and morphology of lipid nanoparticles assessed by cryogenic transmission electron microscopy

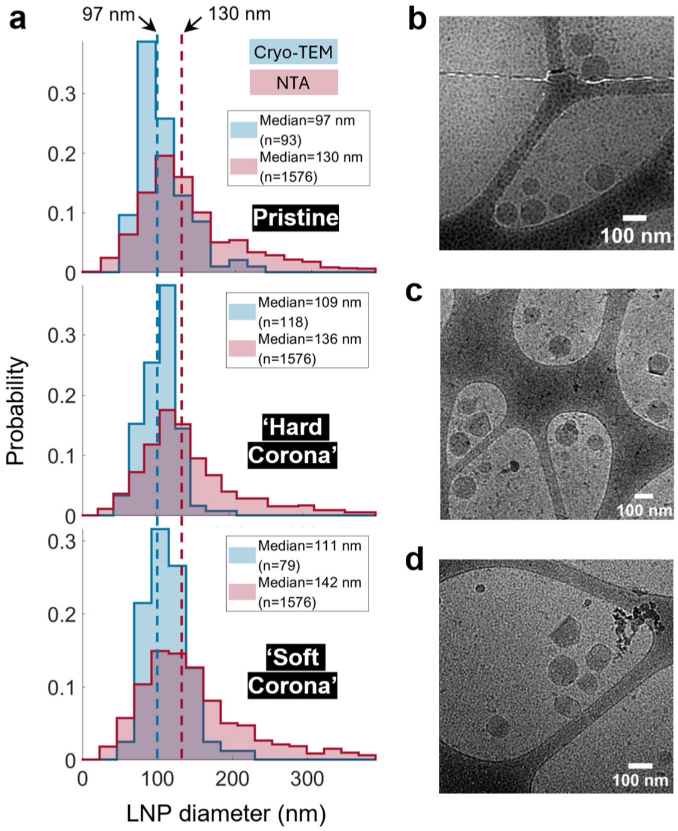

Figure S1. LNPs were formulated using the NanoAssemblr Spark microfluidic mixing system with 100% non-labeled eGFP mRNA and the same lipid composition as used for microscopy in the main text. After dialysis against PBS and Tris buffer containing 8% (w/v) sucrose, mRNA encapsulation efficiency was 90% (RiboGreen assay). Three samples were studied: pristine LNPs (no serum exposure), 'soft corona' LNPs (incubated in 10% FBS for 3h at room temperature, then concentrated), and 'hard corona' LNPs (incubated, concentrated, diluted ~50-fold with Tris buffer, and reconcentrated). To minimize interference from serum-derived particles, FBS was pre-filtered using Pall Nanosep ultrafiltration devices (300 kDa MWCO); the identical devices were used for the concentrating steps in sample preparation. Samples were snap-frozen in liquid nitrogen and kept for approximately 48 hours at -20°C until specimen preparation for cryogenic transmission electron microscopy (cryo-TEM), which was performed using an automated plunge freezer (Leica EM GP) operated at 21°C and >90% humidity. Four µL of sample was applied to glow-discharged, lacey Formvar carbon-coated copper grid (Ted Pella), blotted with filter paper, and vitrified by plunging into liquid ethane. Grids were stored in liquid nitrogen until transfer into the electron microscope using a Fischione Model 2550 cryo-transfer tomography holder. Imaging was performed on a JEOL JEM 2200FS microscope equipped with an in-column Omega energy filter for zero-loss imaging. Micrographs were acquired digitally using a TVIPS F416 camera controlled by SerialEM software under low-dose conditions, with an acceleration voltage of 200 kV and a 10 eV energy-selecting slit.

Panel **(a)** shows size distributions measured with nanoparticle tracking analysis (NTA) and cryo-TEM. Cryo-TEM sizes were manually measured using imageJ; for non-spherical particles, diameter was defined by the largest extension. Vesicle-like structures in the samples (not visible in images) were excluded to focus on LNPs with electron-dense core. Panels **(b–d)** show selected cryo-TEM images of pristine, 'hard corona', and 'soft corona' LNPs, respectively. A small increase in LNP size following serum preincubation is observed for both NTA and cryo-TEM measurements. However, due to the inherent polydispersity of the samples, it remains difficult to assess the statistical significance of the changes. Visual inspection of LNP morphology suggests that serum incubation can alter particle shape and contour, resulting in less spherical particles with disturbed surfaces.

3. Effect of Serum on NeutrAvidin-Biotin-based Lipid Nanoparticle Tethering

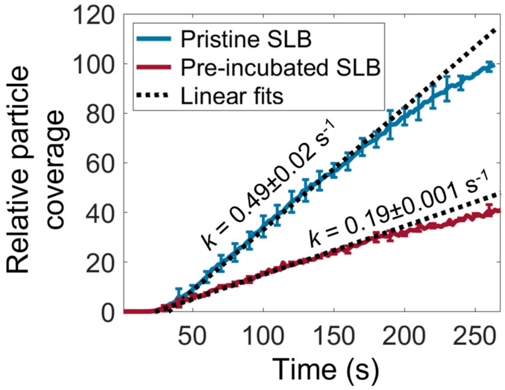

Figure S2. Preincubation of a NeutrAvidin-functionalized anionic supported lipid bilayer (SLB) diminishes the binding rate of Biotin-modified lipid nanoparticles. SLB with POPC, BMP, DOPE-NBD, and DOPE-Cap-biotin at mole percentages of 89.7, 10, 0.25, 0.05, respectively, was formed on a 6 nm nanoporous substrate attached to a sticky-Slide VI 0.15 (ibidi, Germany). In the case of a preincubated SLB, after NeutrAvidin incubation (20 µg/mL, 10 µL/min for 10 min), the SLB was incubated with 10% fetal bovine serum (10 µL/min for 5 min) prior to binding lipid nanoparticles (~5 µg/mL mRNA, 10 µL/min). Binding rates k are estimated by the slopes of linear fits on data (2 replicates), using data within 40 and 120 seconds. The binding rate is reduced by 62 ± 2 % through serum-preincubation.

4. Size Distribution of DMPE-PEG(2000)-ATTO488-labeled Lipid Nanoparticles

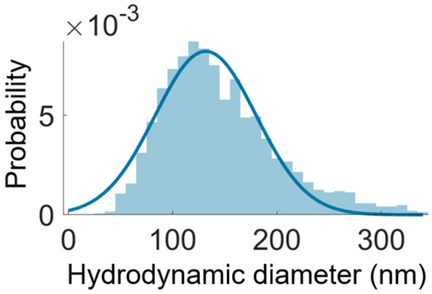

Figure S3. Hydrodynamic diameter distribution measured with nanoparticle tracking analysis of lipid nanoparticles labeled with DMPE-PEG(2000)-ATTO488. Mean and standard deviation of 132 ± 48 nm determined using Gaussian fitting (solid line).
